# The impact of type 1 diabetes on young adults’ health-related quality of life

**DOI:** 10.1186/s12955-020-01370-8

**Published:** 2020-05-12

**Authors:** Madelon B. Bronner, Mariëlle A. C. Peeters, Jane N. T. Sattoe, AnneLoes van Staa

**Affiliations:** 1 Research Centre Innovations in Care, Rotterdam University of Applied Sciences, Rotterdam, The Netherlands; 2 Erasmus School of Health Policy & Management, Erasmus University Rotterdam, Rotterdam, The Netherlands

**Keywords:** Diabetes, Health-related quality of life, Fatigue, Work functioning, Transition to adult care, Young adults

## Abstract

**Background:**

Young adulthood is a challenging period for people with diabetes mellitus type 1 (T1DM) as they are facing multiple life transitions while managing a demanding disease. This poses a risk for impaired health-related quality of life (HRQOL). We assessed HRQOL in a cohort of young adults with T1DM in the Netherlands, and compared outcomes with those of Dutch norm groups of healthy young adults and young adults with a chronic disease.

**Methods:**

We analyzed data collected in a larger evaluation study on transitional care for young adults with T1DM in a nationwide sample in the Netherlands, including twelve participating hospitals. These data had been obtained from online questionnaires completed by young adults with T1DM after they had transferred to adult care. HRQOL was self-reported with the Pediatric Quality of Life Inventory for young adults (PedsQL-YA).

**Results:**

One hundred and sixty-five young adults with T1DM participated (44.2% response); and they scored significantly worse than did healthy peers on all domains of HRQOL, except social functioning. Particularly, functioning at school or work was worse than that of the norm group. The study group’s HRQOL-scores were comparable to norm scores of young adults with chronic diseases, although the physical and social functioning of young people with T1DM was better. One quarter (26.1%) of all young adults with T1DM reported fatigue.

**Conclusions:**

During transition to adulthood, young adults with T1DM struggle to maintain a balance between the demands of managing a disease and their life. Many of them encounter problems at work or school, and suffer from fatigue. These findings underscore the need to regularly assess HRQOL, and to discuss work- and education-related issues in clinical practice.

## Background

Young adults with type 1 diabetes mellitus (T1DM) not only face developmental milestones, but also are expected to take over full responsibility for managing the disease. A high degree of self-control is needed, with the main goal to maintain optimal glycemic control [1]. Optimal glycemic control reduces the risk of disease progression and complications [2, 3]. In an American study on continuous glucose monitoring, only 17% of early young adults (18–25 years) and 30% of late young adults (26–30 years) met recommendations from the American Diabetes Association for glycemic control [4]. Those not meeting the recommendations are at risk for developing diabetes-related complications, like retinopathy or hypertension [2, 5, 6]. These findings taken together make clear that young adulthood represents a critical period for people with T1DM.

Moreover, the process of transition from pediatric to adult health services in this period may result in a gap in services that negatively affects the health of young people with a chronic condition [7]. While multidisciplinary pediatric care for diabetes is often extensive and child-centered, adult care services expect their patients to be more independent and responsible for their own treatment [8]. A review study concluded that more than 25% of young adults had reported a more than 6 months’ gap in medical care during transition to adult health care services [9]. Young adults often show poor clinic attendance or may even become lost to follow-up, which features have been associated with serious and costly medical consequences, such as diabetic ketoacidosis [10]. Therefore, poor transition to adult care may further contribute to adverse health outcomes.

Facing multiple life transitions while coping with a demanding disease can understandably impact one’s health-related quality of life (HRQOL). Previous research has shown that T1DM is associated with impaired HRQOL and loss of utilities [11, 12]. International diabetes guidelines therefore increasingly recommend the use of HRQOL measurement instruments to guide routine care [13]. The vast majority of HRQOL studies so far have been performed in children or adolescents with T1DM [1, 14–16], while reports regarding HRQOL in young adults are scarce [9, 17–19]. In a study in a large global cohort of youth with T1DM (8–25 years), the 19–25 age group reported poorer HRQOL than did the younger age groups [17]. Young adults with T1DM may have age-specific worries that affect their HRQOL, such as concerns about being denied insurance, getting the job they wanted, living independently, future complications and having children [9, 19]. Research on HRQOL in this vulnerable group of young adults is scarce, however, and comparisons with healthy young adults are currently lacking.

The aim of this study was to assess HRQOL in a national cohort of young adults with T1DM in the Netherlands, and to compare their HRQOL scores with those of Dutch norm groups of healthy young adults (aged 18–25) and young adults with different chronic health conditions. This comparison may provide more insight in the impact of T1DM on young adults’ quality of life.

## Methods

### Participants and settings

The data presented in this paper have been collected in a larger evaluation study of transitional care for young adults with T1DM in a nationwide sample in the Netherlands. The study was conducted between April 2016 and October 2018 with the participation of twelve different hospitals, and used a mixed-methods design. The study protocol has been described elsewhere [20]. The present paper deals with the results from an online questionnaire about HRQOL and transfer experiences. Patients were eligible to participate in the study if they had a confirmed diagnosis of T1DM – irrespective of the time elapsed since the diagnosis, – had made the transfer to adult services in 2012–2014, had no cognitive impairment, and were able to speak and read Dutch.

### Measures

Socio-demographic variables collected in the online questionnaire included age, gender, educational level, educational status, employment status and living situation. Educational level was categorized as low (primary education, lower or middle general secondary education), middle (higher secondary education, middle vocational education) and high (higher vocational education, university) education [21]. Educational status was dichotomized as still studying or doing an internship (1) vs. not studying (0). Employment status was dichotomized as having paid work (1) vs. not having paid work (0). Living situation was dichotomized as living independently (1) vs. living with parents (0).

HRQOL was self-reported with the Dutch version of the Pediatric Quality of Life Inventory for young adults (PedsQL-YA) [22]. The scale contains 23 items in four subscales: ‘physical health’, ‘emotional functioning’, ‘social functioning’ and ‘school/work functioning’. Items are scored on a five-point Likert scale from ‘never’ (0) to ‘almost always’ (4). Each answer is reversely scored and rescaled to a 0–100 scale (0 = 100, 1 = 75, 2 = 50, 3 = 25, and 4 = 0). Higher scores indicate better reported HRQOL. Cronbach’s α for the different scales in this study sample ranged from 0.80 to 0.94. Furthermore, minimal clinically important differences range between a 4.4 and 9.1 change in scores on the different scales [23]. Dutch PedsQL-YA norm data of young adults were used for comparisons. In this study population, the self-reported prevalence of chronic health conditions was 21.1%. Most common conditions were asthma (34.3%), psychiatric disorders (10.9%), digestive disorders and gastrointestinal diseases (10.2%), and skin diseases (5.8%) [24].

### Design and procedure

In January 2018, the twelve participating hospitals invited via email eligible patients who had made the transfer to adult services in 2012, 2013 or 2014 to complete an online questionnaire. Five of these hospitals also invited patients who had made the transfer in 2015 and 2016. Reminders were sent by email after two and four weeks. To boost participation, every third respondent was to receive a €20 gift voucher. Those who eventually participated provided online consent to use the collected data for scientific research. The Medical Ethics Review Board of Erasmus MC approved the original study protocol (MEC-2014-246), and ethical approval was obtained from all local hospital review boards.

### Statistical analyses

All analyses were conducted using SPSS 22.0 [25]. First, preparatory and descriptive analyses were performed (e.g. scale scores, Cronbach’s alpha, distributions of scores, socio-demographics, effect sizes [26]). Second, differences on socio-demographics (age and gender) between non-responders and comparison groups were calculated using t-test, ANOVA, and Chi-square (χ^2^) tests. Next, differences on the PedsQL-YA scale scores between the study group and norm groups (healthy and chronic) were examined with multiple regression analysis, corrected for age and gender. These are potential confounders for HRQOL [24]. The group variable (T1DM, healthy, chronic) were coded into dummy variables with diabetes as reference group. Preliminary analyses were conducted to ensure no violation of the assumptions of normality, linearity, multicollinearity and homoscedasticity. Then, a regression model for the PedsQL-YA total scale was built to compare group differences, using the T1DM group as reference group, and age and gender as confounders. Subsequently, multivariate regression analyses were performed for PedsQL-YA subscales.

Exploratory analyses were performed on HRQOL-scores by examining on which items the study group and the norm groups reported most problems. Chi-square tests were used to analyze differences on the items between the study group and the norm groups.

## Results

Three hundred seventy-three eligible patients with T1DM were invited to fill out the online questionnaire, of whom 165 completed the questionnaire (response rate 44.2%). Their mean age was 22.6 years (SD = 1.6, range 19.0–28.0) and 60.0% was female. Most (69.5%) had middle level education and a paid job (78.0%). Half of the whole group was still studying or had an internship, and 61.0% lived with their parents (Table 1). The mean age at transfer was 18.4 years (SD = 1.2); the mean time elapsed between transfer and completing the questionnaire was 4.7 years (SD = 1.1). Non-response data were available only with regard to gender and age for young adults who had made the transfer to adult services in 2012–2014. Data showed that 36.9% of the non-responders was female and had an average age of 23.6 (SD = 1.4). Thus, non-responders were more often male (χ^2^ = 21.814, *p* < 0.000) and statistically significantly older than the participating young adults (t = 6.337 p < 0.000).

**Table 1:** Characteristics of 165 young adults with T1DM participating in the study

	N (%)
Age, years ± SD	22.7 ± 1.6
Female	99 (60.0)
Education	
Low	12 (7.3)
Middle	115 (69.7)
High	38 (23.0)
Studying or internship	83 (50.3)
Paid work	128 (77.6)
Living with parents	99 (60.0)

Dutch PedsQL-YA norm data of 310 healthy young adults and 75 young adults with a chronic disease were used for comparisons. The average age of the healthy group was 22.2 (SD = 2.4); 51.3% was female. In the chronic disease group the average age was 22.0 (SD = 2.4); 62.7% was female. Age (F = 2.740, df = 2, *p* = 0.065) and gender (χ^2^ = 5.135, *p* = 0.077) did not significantly differ from our study group (Table 2).

**Table 2:** PedsQL-YA scores and effect sizes (Cohen’s *d*) for young adults with T1DM compared to two norm groups (healthy and chronic disease) for age group 18–25

	Diabetes	Healthy		Chronic Disease	
	*N* = 165	*N* = 310		*N* = 75	
	*M* (*SD*)	*M* (*SD*)	*d*	*M* (*SD*)	*d*
Total	79.0 (15.3)	85.9 (11.2)	0.6	75.2 (15.1)	− 0.2
Physical	84.5 (15.9)	90.2 (12.5)	0.5	77.8 (20.5)	−0.3
Emotional	70.9 (21.9)	78.4 (17.7)	0.4	69.7 (18.1)	−0.1
Social	86.2 (15.1)	88.4 (13.7)	0.2	79.2 (17.4)	−0.4
School	71.0 (19.0)	84.0 (14.3)	0.9	72.7 (16.9)	0.1

Note. Higher scores indicate better HRQOL; Norm data (healthy and chronic disease) taken from Limperg et al. [24]

Multivariate regression analyses were used to compare HRQOL-scores between the different groups, controlling for gender and age. The model for the total PedsQL-score was statistically significant, *F* (4, 545) = 19.237, *p* < 0.000, and accounted for 12.4% of the variance. The T1DM study group had significantly different HRQOL-scores compared to both norm groups (healthy and chronic disease). HRQOL-scores in the study group were lower than in the healthy norm group (*β =* 0.228*, p* = 0.000), and higher than in the chronic disease group (*β =* − 0.101*, p* = 0.026). This difference (− 4.015) was too small to be of clinical relevance. Age (*β =* **−** 0.085*, p* = 0.036) and gender (*β =* − 0.148*, p* = 0.000) were significantly related to HRQOL. Males and younger participants had better HRQOL-scores.

To further explore the differences on HRQOL scores, MUltivariate regression analyses were used to compare HRQOL-scores between the different groups, controlling for gender and age. Data are presented in Table 3. Compared to their healthy peers, young adults with T1DM scored significantly worse on all domains of HRQOL except social functioning. Particularly, functioning at school or work was worse than that of the healthy norm group. This difference (− 13.0) could be considered clinically significant. The HRQOL-scores of the T1DM study group were comparable to the scores of the chronic disease group on emotional and school/work functioning. Scores on physical and social functioning were significantly better than the norm scores of young adults with a chronic disease. However, these statistically significant differences were of minimal clinical relevance.

**Table 3:** Multivariate regression analysis for groups comparisons (diabetes, healthy and chronic disease group) on the PedsQL-YA scales, corrected for age and gender

		Unstandardized coefficients		Standardized coefficients		
		B	SE	β	t	*p*-value
Total	Diabetes	reference				
	Healthy	6.294	1.248	0.228	5.042	0.000
	Chronic disease	−4.015	1.801	−0.101	−2.229	0.026
	Age	−0.533	0.254	−0.085	−2.103	0.036
	Gender	−4.069	1.116	−0.148	−3.645	0.000
Physical	Diabetes	reference				
	Healthy	5.007	1.411	0.161	3.549	0.000
	Chronic disease	−6.986	2.035	−0.155	−3.433	0.001
	Age	−0.682	0.287	−0.097	−2.379	0.018
	Gender	−4.862	1.261	−0.157	−3.854	0.000
Emotional	Diabetes	reference				
	Healthy	6.399	1.794	0.163	3.568	0.000
	Chronic disease	−1.484	2.587	−0.026	−0.573	0.567
	Age	−0.875	0.364	−0.098	−2.402	0.017
	Gender	−8.592	1.604	−0.220	−5.357	0.000
Social	Diabetes	reference				
	Healthy	1.938	1.418	0.064	1.367	0.172
	Chronic disease	−7.140	2.045	−0.164	−3.491	0.001
	Age	−0.237	0.288	−0.035	−0.822	0.411
	Gender	−1.797	1.268	−0.060	−1.417	0.157
School	Diabetes	reference				
	Healthy	12.742	1.571	0.366	8.111	0.000
	Chronic disease	1.459	2.266	0.029	0.644	0.520
	Age	−0.251	0.319	−0.032	−0.788	0.431
	Gender	−0.606	1.405	−0.017	−0.432	0.666

Note. Higher scores indicate better HRQOL; Norm data (healthy and chronic disease) taken from Limperg et al. [24]; Gender is coded as male = 0;female = 1

The young adults with diabetes experienced most problems on the following three PedsQL-YA items: ‘I have low energy’, ‘I forget things’ and ‘It is hard to pay attention at work or study’. More than a quarter (26.1%) of the young adults with diabetes was ‘almost always’ or ‘often’ low in energy, 17.0% forgot things and 13.3% had a hard time paying attention. In comparison with the healthy norm group, relatively more young adults with T1DM reported low energy (healthy norm: 4.8%; χ^2^ = 45.242, *p* = 0.000), forgot things (healthy norm: 1.6%; χ^2^ = 39.283, p = 0.000), and had problems paying attention (healthy norm: 4.5%; χ^2^ = 11.952, *p* = 0.001). There were no significant differences between the T1DM group and the chronic disease group.

## Discussion

This unique nationwide study in the Netherlands shows that the surveyed young adults with T1DM had a good social life, but performed worse on physical, emotional, and school/work functioning than do their healthy counterparts. Particularly functioning at school or work was impaired. HRQOL of young adults with T1DM was comparable to norm scores of young adults with chronic diseases, as the differences were too small to be of clinical relevance. In addition, a quarter of young adults with T1DM reported fatigue.

Earlier studies in young adults with chronic diseases also showed the negative impact of the condition on social participation and work. For example, Sattoe et al. [27] found four patterns of social participation among young adults with a chronic condition. Those with a social participation pattern similar to that of healthy age-mates reported lower HRQOL. Keeping up with social demands might be challenging for young adults with a chronic condition. A recent review on the impact of growing up with a chronic disease on psychosocial outcomes showed a lower likelihood of having a paid job [28]. Similarly, a study in adults with T1DM showed higher unemployment and sick leave rates among this group compared to the general population – while they were slightly better educated [9, 29]. Extra guidance for finding the right balance between social life and work could be beneficial for these young adults.

One quarter of our study population reported fatigue, compared to only 4.8% of the healthy norm group. Fatigue is a prevalent and burdensome complaint of patients with T1DM [30, 31], and is generally found in childhood chronic disease [32]. Research shows that these patients’ fatigue is not simply explained by somatic processes such as suboptimal glycemic control, but that cognitions and behaviors also play an important role in the perpetuation of fatigue [31]. Therefore, young adults with T1DM could probably benefit from cognitive-behavioral therapy (CBT) to manage their fatigue – as shown in a large multicenter, randomized controlled trial [30].

All in all, growing into adulthood with T1DM may go hand-in-hand with impaired HRQOL and with fatigue. Young adulthood represents a vulnerable period with high health risks, even higher than in childhood or adolescence. This is confirmed by a large global study among youth (8–25 years) with T1DM, in which the young adult age group reported the lowest HRQOL [17]. Therefore, it is important to monitor their HRQOL from childhood into adulthood. Regular assessments in outpatient clinics provide the opportunity to discuss health-related topics [33], like functioning at school or work and to detect problems during transition. A smooth transition to adult health care services – preferably with the use of a structured transition program – is critical [34]. However, the appropriate ingredients and outcomes of such a program have not yet been detailed [35]. In a study of Fair et al. [36], achieving optimal quality of life was rated as the most important outcome for successful transition.

The present study has several strengths, including the relatively large sample size, the comparison with Dutch norm scores for the PedsQL-YA controlling for age and gender, a nationwide representation of young adults with T1DM, and the bridging of a significant gap in knowledge on quality of life among young adults with T1DM. Limitations include the suboptimal response rate (44.2%), although this is comparable with that in other post-transition diabetes studies [37], as well as the significant differences in gender and age between responders and non-responders. Non-responders were more often male and significantly older than respondents. As men tend to report higher HRQOL [24], this could have led to an overestimation of problems in HRQOL. However, health surveys in adolescents generally show that non-response bias leads to a substantial underestimation of health problems [38]. Therefore, it is difficult to estimate the size and the direction of non-response bias in our sample. Furthermore, the cross-sectional design precluded us from examining causality. Longitudinal studies are necessary to understand the causal underpinnings of HRQOL. Additionally, we did not study what variables contributed to better or worse HRQOL. Our primary aim was to explore the impact of T1DM on young adults’ quality of life. For future studies, examining predictors for HRQOL would be of interest. Candidate predictors include worries about the future, level of physical activity, and clinical parameters such as HbA1c, BMI, time-in-range, and fear of complications like hypoglycemia [19]. More research on chronic fatigue in T1DM is also needed, as this is an understudied complaint [39].

## Conclusion

The impact of T1DM on young adults’ quality of life is substantial. These young adults with T1DM may be socially active, but as a downside they may suffer from fatigue and experience problems at work. Finding the right balance between personal and professional life while managing a demanding disease is not easy for these young adults. This underscores the desirability of regular assessing HRQOL, including work- and study-related issues, in clinical practice. Particularly, functioning at school or work was worse than that of the norm group.

## Data Availability

Anonymized datasets can be made available on reasonable request to the corresponding author.

## Abbreviations

HRQOL: Health-related quality of life
T1DM: Type 1 diabetes mellitus

## References

[1] Murillo M, Bel J, Perez J, Corripio R, Carreras G, Herrero X (2017). Health-related quality of life (HRQOL) and its associated factors in children with type 1 diabetes mellitus (T1DM). BMC Pediatr.

[2] Bryden KS, Dunger DB, Mayou RA, Peveler RC, Neil HA (2003). Poor prognosis of young adults with type 1 diabetes: a longitudinal study. Diabetes Care.

[3] DiMeglio LA, Acerini CL, Codner E, Craig ME, Hofer SE, Pillay K (2018). ISPAD clinical practice consensus guidelines 2018: glycemic control targets and glucose monitoring for children, adolescents, and young adults with diabetes. Pediatr Diabetes.

[4] Beck RW, Calhoun P, Kollman C (2012). Use of continuous glucose monitoring as an outcome measure in clinical trials. Diabetes Technol Ther.

[5] Dabelea D, Stafford JM, Mayer-Davis EJ, D'Agostino R, Dolan L, Imperatore G (2017). Association of type 1 diabetes vs type 2 diabetes diagnosed during childhood and adolescence with complications during teenage years and young adulthood. JAMA.

[6] James S, Gallagher R, Dunbabin J, Perry L (2014). Prevalence of vascular complications and factors predictive of their development in young adults with type 1 diabetes: systematic literature review. BMC Res Notes.

[7] Campbell F, Biggs K, Aldiss SK, O'Neill PM, Clowes M, McDonagh J (2016). Transition of care for adolescents from paediatric services to adult health services. Cochrane Database Syst Rev.

[8] Viner RM (2008). Transition of care from paediatric to adult services: one part of improved health services for adolescents. Arch Dis Child.

[9] Monaghan M, Helgeson V, Wiebe D (2015). Type 1 diabetes in young adulthood. Curr Diabetes Rev.

[10] Mazur A, Dembinski L, Schrier L, Hadjipanayis A, Michaud PA (2017). European academy of Paediatric consensus statement on successful transition from paediatric to adult care for adolescents with chronic conditions. Acta Paediatr.

[11] Braga de Souza AC, Felicio JS, Koury CC, Neto JF, Mileo KB, Santos FM (2015). Health-related quality of life in people with type 1 Diabetes Mellitus: Data from the Brazilian Type 1 Diabetes Study Group. Health Qual Life Outcomes.

[12] Smith-Palmer J, Bae JP, Boye KS, Norrbacka K, Hunt B, Valentine WJ (2016). Evaluating health-related quality of life in type 1 diabetes: a systematic literature review of utilities for adults with type 1 diabetes. Clinicoecon Outcomes Res.

[13] Delamater AM, de Wit M, McDarby V, Malik J, Acerini CL (2014). International Society for P, et al. ISPAD Clinical Practice Consensus Guidelines 2014. Psychological care of children and adolescents with type 1 diabetes. Pediatr Diabetes.

[14] Cruz D, Collet N, Nobrega VM (2018). Quality of life related to health of adolescents with DM1: an integrative review. Cien Saude Colet.

[15] Lukacs A, Mayer K, Sasvari P, Barkai L (2018). Health-related quality of life of adolescents with type 1 diabetes in the context of resilience. Pediatr Diabetes.

[16] Nieuwesteeg A, Pouwer F, van der Kamp R, van Bakel H, Aanstoot HJ, Hartman E (2012). Quality of life of children with type 1 diabetes: a systematic review. Curr Diabetes Rev.

[17] Anderson BJ, Laffel LM, Domenger C, Danne T, Phillip M, Mazza C (2017). Factors associated with diabetes-specific health-related quality of life in youth with type 1 diabetes: the global TEENs study. Diabetes Care.

[18] Varni JW, Delamater AM, Hood KK, Raymond JK, Driscoll KA, Wong JC (2018). Diabetes symptoms predictors of health-related quality of life in adolescents and young adults with type 1 or type 2 diabetes. Qual Life Res.

[19] Kent DA, Quinn L (2018). Factors that affect quality of life in young adults with type 1 diabetes. Diabetes Educ.

[20] Sattoe JN, Peeters MA, Hilberink SR, Ista E, van Staa A (2016). Evaluating outpatient transition clinics: a mixed-methods study protocol. BMJ Open.

[21] Schneider, SL. The International Standard Classification of Education 2011. In: Birkelund, GE. Class and Stratification Analysis. Emerald Group Publishing Ltd. 2013:365–79.

[22] Varni JW, Limbers CA (2009). The PedsQL 4.0 generic Core scales Young adult version: feasibility, reliability and validity in a university student population. J Health Psychol.

[23] Varni JW, Burwinkle TM, Seid M, Skarr D (2003). The PedsQL 4.0 as a pediatric population health measure: feasibility, reliability, and validity. Ambul Pediatr.

[24] Limperg PF, Haverman L, van Oers HA, van Rossum MA, Maurice-Stam H, Grootenhuis MA (2014). Health related quality of life in Dutch young adults: psychometric properties of the PedsQL generic core scales young adult version. Health Qual Life Outcomes.

[25] Field AP. Discovering statistics using IBM SPSS. 5th Edition. Sage Publications Ltd. 2018.

[26] Cohen J. Statistical power analysis for the behavioral sciences. Hillsdale, N.J: L. Erlbaum Associates; 1988.

[27] Sattoe JN, Hilberink SR, van Staa A, Bal R (2014). Lagging behind or not? Four distinctive social participation patterns among young adults with chronic conditions. J Adolesc Health.

[28] Maurice-Stam H, Nijhof SL, Monninkhof AS, HSA H, Grootenhuis MA. Review about the impact of growing up with a chronic disease showed delays achieving psychosocial milestones. Acta Paediatr. 2019;108(12):2157–69. 10.1111/apa.14918 31250466

[29] Nielsen HB, Ovesen LL, Mortensen LH, Lau CJ, Joensen LE (2016). Type 1 diabetes, quality of life, occupational status and education level - a comparative population-based study. Diabetes Res Clin Pract.

[30] Menting J, Tack CJ, van Bon AC, Jansen HJ, van den Bergh JP, Mol M (2017). Web-based cognitive behavioural therapy blended with face-to-face sessions for chronic fatigue in type 1 diabetes: a multicentre randomised controlled trial. Lancet Diabetes Endocrinol.

[31] Menting J, Tack CJ, Donders R, Knoop H (2018). Potential mechanisms involved in the effect of cognitive behavioral therapy on fatigue severity in type 1 diabetes. J Consult Clin Psychol.

[32] Nap-van der Vlist MM, Dalmeijer GW, Grootenhuis MA, van der Ent CK, van den Heuvel-Eibrink MM, Wulffraat NM, et al. Fatigue in childhood chronic disease. Arch Dis Child. 2019;104(11):1090–95. 10.1136/archdischild-2019-316782 31175124

[33] Haverman L, Limperg PF, Young NL, Grootenhuis MA, Klaassen RJ (2017). Paediatric health-related quality of life: what is it and why should we measure it?. Arch Dis Child.

[34] Schultz AT, Smaldone A (2017). Components of interventions that improve transitions to adult care for adolescents with type 1 diabetes. J Adolesc Health.

[35] Sattoe JNT, Hilberink SR, van Staa A (2017). How to define successful transition? An exploration of consensus indicators and outcomes in young adults with chronic conditions. Child Care Health Dev.

[36] Fair C, Cuttance J, Sharma N, Maslow G, Wiener L, Betz C (2016). International and interdisciplinary identification of health care transition outcomes. JAMA Pediatr.

[37] Garvey KC, Finkelstein JA, Laffel LM, Ochoa V, Wolfsdorf JI, Rhodes ET (2013). Transition experiences and health care utilization among young adults with type 1 diabetes. Patient Prefer Adherence.

[38] Cheung KL, Ten Klooster PM, Smit C, de Vries H, Pieterse ME (2017). The impact of non-response bias due to sampling in public health studies: a comparison of voluntary versus mandatory recruitment in a Dutch national survey on adolescent health. BMC Public Health.

[39] Jensen O, Bernklev T, Jelsness-Jorgensen LP (2017). Fatigue in type 1 diabetes: a systematic review of observational studies. Diabetes Res Clin Pract.

